# Initial and Sustained Participation in an Internet-delivered Long-term Worksite Health Promotion Program on Physical Activity and Nutrition

**DOI:** 10.2196/jmir.1788

**Published:** 2012-03-05

**Authors:** Suzan JW Robroek, Dennis EM Lindeboom, Alex Burdorf

**Affiliations:** ^1^Department of Public HealthErasmus MCRotterdamNetherlands; ^2^LifeguardUtrechtNetherlands

**Keywords:** Participation, Retention, Internet, Physical activity, Nutrition, Workplace, Health promotion

## Abstract

**Background:**

Determinants of participation in health promotion programs are largely unknown. To evaluate and implement interventions, information is needed regarding their reach as well as regarding the characteristics of program users and non-users.

**Objective:**

In this study, individual, lifestyle, and health indicators were investigated in relation to initial, and sustained participation in an Internet-delivered physical activity and healthy nutrition program in the workplace setting. In addition, determinants of program website use were studied.

**Methods:**

Determinants of participation were investigated in a longitudinal study among employees from six workplaces participating in a two-year cluster randomized controlled trial. The employees were invited by email to participate. At baseline, all participants visited a website to fill out the questionnaire on lifestyle, work, and health factors. Subsequently, a physical health check was offered, followed by face-to-face advice. Throughout the study period, all participants had access to a website with information on lifestyle and health, and to fully automated personalized feedback on the questionnaire results. Only participants in the intervention received monthly email messages to promote website visits during the first year and had access to additional Web-based tools (self-monitors, a food frequency questionnaire assessing saturated fat intake, and the possibility to ask questions) to support behavior change. Website use was monitored by website statistics measuring access. Logistic regression analyses were conducted to identify characteristics of employees who participated in the program and used the website.

**Results:**

Complete baseline data were available for 924 employees (intervention: n=456, reference: n=468). Lifestyle and health factors were not associated with initial participation. Employees aged 30 years and older were more likely to start using the program and to sustain their participation. Workers with a low intention to increase their physical activity level were less likely to participate (Odds Ratio (OR)=0.60, 95% Confidence interval (95%CI), 0.43-0.85) but more likely to sustain participation throughout the study period (ORs ranging from 1.40 to 2.06). Furthermore, it was found that smokers were less likely to sustain their participation in the first and second year (OR=0.54, 95%CI 0.35-0.82) and to visit the website (OR=0.72, 95%CI 0.54-0.96). Website use was highest in the periods immediately after the baseline (73%) and follow-up questionnaires (71% and 87%). Employees in the intervention were more likely to visit the website in the period they received monthly emails (OR=5.88, 95%CI 3.75-9.20) but less likely to visit the website in the subsequent period (OR=0.62, 95%CI 0.45-0.85).

**Conclusions:**

Modest initial participation and high attrition in program use were found. Workers with a low intention to change their behavior were less likely to participate, but once enrolled they were more likely to sustain their participation. Lifestyle and health indicators were not related to initial participation, but those with an unhealthy lifestyle were less likely to sustain. This might influence program effectiveness. Regular email messages prompted website use, but the use of important Web-based tools was modest. There is a need for more appealing techniques to enhance retention and to keep those individuals who need it most attracted to the program.

**Trial Registration:**

ISRCTN52854353; http://www.controlled-trials.com/ISRCTN52854353

## Introduction

Low participation is a concern in health promotion programs [1]. The workplace has been thought to be a promising setting for health promotion, providing access to large groups. However, in workplace health promotion, participation levels are typically below 50% [1]. These low participation levels may have important consequences for the effectiveness of health promotion programs and raise concerns about the generalizability of results. Therefore, information on participation and its determinants is needed. Furthermore, to assess whether participants obtain sufficient exposure to the intervention, program use as well as characteristics of program users and non-users need to be studied [2].

The RE-AIM framework stresses the importance of evaluating the reach of health promotion programs and the representativeness of program participants [3]. Several studies provide information on the reach of their program, but information on the representativeness of program participants, or specifically the reach of the program to individuals at-risk, is scarce [1]. In addition to program reach and determinants of initial participation, sustained participation is of importance because studies with longer-term program utilization tend to have better outcomes concerning physical activity (PA), dietary behavior change [4], and weight loss [5]. In our study, a health promotion program was offered in the workplace setting, combining face-to-face contact with an Internet-delivered program. It has been found that frequent email messages might enhance sustained participation in Internet-delivered programs [6,7]. However, low initial participation levels and high levels of attrition are also common in Internet-delivered health promotion programs [4,8,9]. 

In previous research, several factors that influence participation in Internet-delivered behavior change programs have been identified. It has been reported that women [10-14], and individuals with a medium or high educational level [10,12-13] are more likely to start participating. However, there is only scarce information on lifestyle- and health-related determinants of initial participation, with some studies showing that individuals with a normal body weight or a healthier lifestyle more often participate [14,15], and another study reporting that individuals who needed it most were reached [16].

A number of studies on determinants of sustained participation reported that particularly women [14,17], individuals with a lower educational level [18], and older employees [10,13,14,16,17,19,20] were more likely to sustain participating in Internet-delivered behavior change programs. For lifestyle and health indicators, the evidence is contradictory. In some studies, participants with a healthy lifestyle at baseline, particularly non-smokers, more often sustained their participation [10,19,21], whereas in other studies a higher sustained participation was present among overweight participants [10] or among those not complying with healthy lifestyle guidelines [14].

Since exposure to behavior change programs is required for effective interventions, insight into program use and into characteristics of program users and non-users is needed. More studies are needed to investigate whether there is a consistent pattern in whom we reach in health promotion and who keeps participating in primary preventive interventions. Therefore, the aims of this study were to investigate in an occupational population participation in an Internet-delivered health promotion program on PA and healthy nutrition and to identify the factors that determined their participation.

## Methods

### Study Design, Participants and Recruitment

**Figure 1:**
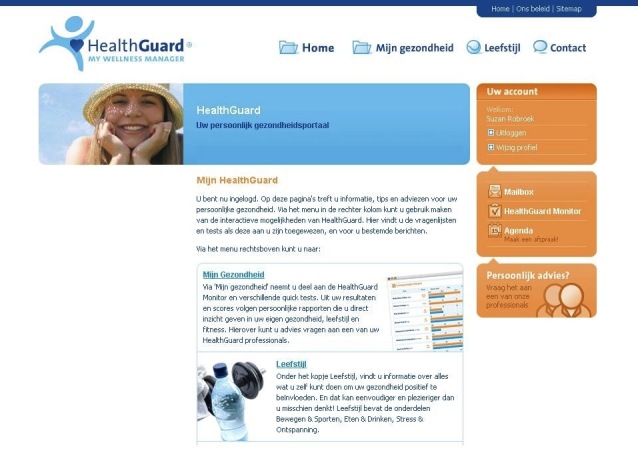
Screenshot of the website.

An observational study was conducted among participants who had enrolled in a 2-year cluster randomized controlled trial (cRCT), with departments (n=74) within companies (n=6) as the unit of randomization. Participants were blinded to the type of intervention. An extensive description of the cRCT conducted between November 2007 and October 2010 is published elsewhere [22]. Participants were employees from health care organizations (n=2), commercial services (n=2), and the executive branch of government (n=2) in the Netherlands. Within the participating companies, the study was announced through email, intranet, and/or a company magazine. Afterwards, an invitation to participate, with login codes, were sent by the provider of the website (Lifeguard Inc.). Participants enrolled voluntarily in the study by visiting the website (see Figure 1) and completing the online baseline questionnaire on lifestyle factors, health, and work demands. The website also provided general information concerning lifestyle and health, as well as feedback based on the online questionnaire, and could be visited from every computer. Subsequently, all participants could participate in a physical health check followed by a face-to-face contact discussing the health check and questionnaire results. These health checks and face-to-face contacts took place at the company. One year after the baseline measurements, participants were asked to fill out the first follow-up questionnaire. Two years after baseline, all participants were invited to fill out the second follow-up questionnaire and to participate again in the physical health check.

In total, 12,895 employees were invited to participate. The three largest companies restricted the maximum number of participants to 200 or 300 on a ‘first in’ principle. In the companies not restricting the enrolment, participation levels ranged from 36% to 61%. During the first year after baseline, 860 non-participants received an abbreviated version of the questionnaire asking for the reason for not participating and a few questions on lifestyle, health, and work. A sample of non-participants in the health care organizations, and all non-participants in the 2 commercial services and in 1 executive branch of government received the questionnaire. In the other organization in the executive branch of government, non-participants were not invited to fill out the questionnaire. Since the program was initiated in the holiday period and communicated in a very limited way, and only 200 workers were allowed to participate, most workers in that organization were unaware of the program. Due to privacy regulations, the non-participant questionnaire was sent out only once without any reminders.

The Medical Ethics Committee of Erasmus MC, University Medical Center in Rotterdam, the Netherlands, approved the study and all participants gave written informed consent at the face-to-face contact.

#### Reference Condition

Participants in the reference group had access to individual reports based on the online questionnaire and physical health check. These reports presented details on their personal PA level and fruit and vegetable intake, recommended levels of physical activity and fruit and vegetable intake in the Netherlands, and references to the general information on lifestyle and health on the website.

#### Intervention Condition

**Figure 2:**
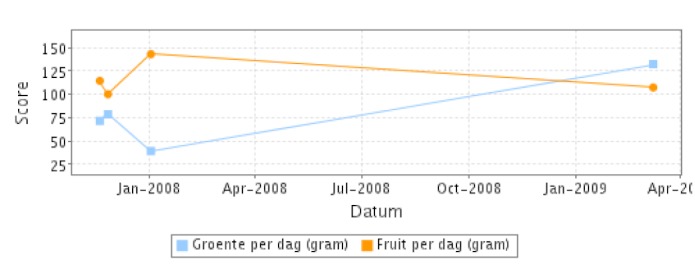
Example of the self-monitor for fruit and vegetable intake.

Participants in the intervention condition had access to several additional Web-based tools compared to participants in the reference condition. The following Web-based tools were available during the two-year study period. Participants in the intervention condition had access to a more extensive computer-tailored advice on their self-reported PA and nutrition behavior than participants in the reference condition. The electronically generated advice included personal and action feedback taking into account perceived barriers for participants not meeting the guidelines [22,23]. Secondly, online self-monitors were available to monitor progress on fruit and vegetable intake, PA, and weight and to obtain tracking charts (see Figure 2). Thirdly, participants in the intervention condition were invited to fill out a food frequency questionnaire (FFQ) assessing saturated fat intake for tailored advice [24]. Furthermore, they had the opportunity to asking questions to several professionals. In addition, during the first 12 months of the study, monthly email messages were sent on PA and nutrition, and participants were encouraged to make use of the available Web-based tools on their personalized web page.

#### Initial and Sustained Participation

Initial participation was defined as filling out the baseline questionnaire to obtain advice on lifestyle. Sustained participation was defined as filling out the questionnaire after 12 or 24 months. The use of the Internet-delivered program was measured as visiting the website throughout the study period. To correct for website visit solely to fill out questionnaires, one login was subtracted in the periods following an invitation to fill out the questionnaires. Two periods for program use were distinguished: the first three months after filling out the questionnaire, and the remaining period.

### Determinants

#### Individual Characteristics

In the baseline questionnaire, participants were asked about their age, sex, education, ethnicity, and marital status. Educational level was assessed by the highest level of education completed and was defined as low (primary school, lower and intermediate secondary school, or lower vocational training), intermediate (higher secondary school or intermediate vocational school) and high (higher vocational school or university). Ethnicity distinguished Dutch born citizens from others, according to the standardized procedures described by Statistics Netherlands [25].

#### Lifestyle and Health Indicators

Moderate and vigorous intensity PA was self-assessed in the baseline questionnaire by the short version of the International Physical Activity Questionnaire (IPAQ) [26]. The average time spent on PA per day was calculated. Walking was not included in this calculation, because casual walking is regarded as a light-intensity activity [27]. For all behaviors, a dichotomous variable was calculated. For PA level, we used a cut-off point of 30 minutes or more of moderate or vigorous PA per day. For fruit and vegetable intake (FV), 400 grams of fruit and vegetable intake as measured with a self-administered 9-item validated Dutch Food Frequency Questionnaire was used as cut-off point [28]. Smoking was defined as current smoking status, and excessive alcohol use as drinking 15 or more glasses of alcohol per week for women and 22 or more glasses for men. The first question of the Short Form-12 questionnaire rated perceived general health, which was dichotomized into ‘poor or moderate’ and ‘good to excellent’ [29].

In the physical health check, height and weight were measured to calculate the Body Mass Index (BMI) and to categorize individuals as normal weight (BMI<25kg/m^2^), overweight (25£BMI<30kg/m^2^), and obese (BMI³30kg/m^2^). For non-participants, weight was self-reported in the questionnaire. Total blood cholesterol was measured in non-fasting blood through a finger prick (Accutrend GC, Roche Company, Mannheim, Germany), and blood pressure was measured with a fully automated sphygmomanometer (Omron M4-I, Omron HealthCare Europe BV, Hoofddorp, the Netherlands). A total cholesterol level above 5.0 mmol/l and a systolic or diastolic blood pressure above 140 mmHg and 90 mmHg respectively were considered elevated. A submaximal exercise test on a bicycle ergometer was conducted to predict maximal oxygen uptake, according to the American College of Sports Medicine’s protocol, using their sex- and age-dependent cut-off points [30].

#### Social Cognitive Variables

For both PA and fruit and vegetable intake, attitude, social support, self-efficacy, and intention to change were measured in the baseline questionnaire. To measure attitude, individuals were asked to indicate on a 5-point Likert scale (‘certainly not’ to ‘certainly’) whether they thought improving the behavior would take a lot of effort [31]. Those participants who answered ‘probably or certainly not’ were considered as having a positive attitude. Social support was measured by asking whether family and friends support them in changing the specific behaviors (4-point Likert scale ranging from ‘seldom’ or ‘never’ to ‘a lot’) [32]. High social support was defined as perceiving ‘pretty much’ or ‘a lot’ of support. Self-efficacy was assessed on a 5-point Likert scale (‘certainly not’ to ‘certainly’) by asking whether the participant was confident to engage in the healthy behaviors in the next month [32]. High self-efficacy was defined as ‘probably’ or ‘certainly’ confident to change the behavior. Intention was also measured on a 5-point Likert scale (‘certainly not’ to ‘certainly’) by asking whether the participant intended to change the behavior in the next month [29]. A high intention was defined as ‘probably’ or ‘certainly’ intended to change the behavior.

#### Work-Related Factors

Physical work demands were measured by one item, asking whether participants perceive their current job as mainly physically or mentally demanding.

### Statistical Analyses

Descriptive statistics were used to present the baseline characteristics of the study population. For *initial participation*, individual characteristics, behaviors, social cognitive variables, and health indicators of participants were compared with workers who did not start in the program. Determinants of initial participation were investigated with univariate logistic regression analyses.

For *sustained participation*, individual characteristics, behaviors, and health indicators from participants at 12 and 24 months follow-up were compared with initial participants who dropped out of these follow-up measurements. Determinants of sustained participation were also investigated with logistic regression analyses. First, univariate logistic regression models were carried out to determine the single effects of the possible determinants. All variables with a *p*-value less than 0.05 in the univariate model either at 12 or 24 months were included in both multivariate analyses to increase comparability between models. A backward selection method was used to determine the multivariate models, whereby age and sex were included by default. Variables with a *p*-value of 0.05 or less in either the 12 months or 24 months model were retained in the multivariate model. All analyses were adjusted for company and carried out with the PASW Statistics version 17.0.2 (SPSS Inc, Chicago, IL, USA).

For *website use* individual characteristics, behaviors, and health indicators from participants who visited the Internet-delivered program were compared with non-visitors. Descriptive statistics presented the use of the different website functionalities, and multilevel General Estimating Equations (GEE) were used for determinants of website visit. The same procedure was followed as for sustained participation. GEE is suitable for the analysis of repeated measurements within participants and was carried out with SAS 9.2 statistical software package.

The results are presented by the odds ratios (OR) and corresponding 95% confidence intervals (95%CI), with ORs below and above 1 representing respectively lower and higher participation.

## Results

At baseline, 987 participants filled out the questionnaire, of which 36 were excluded due to working less than 12 hours per week for the company, and an additional 27 were excluded because they did not complete the questionnaire. In total, 924 employees met the inclusion criteria. Concerning the non-participants, 213 employees out of the 860 invited non-participants responded (24.8%) of which 183 (85.9%) met the inclusion criteria. The baseline characteristics of the study group are presented in Table 1. Half of the participants (450 out of 924, 49%) were male workers. The mean age was 42 years, ranging from 20 to 63 years, and 414 out of 924 (45%) had a high education level. Almost a third of the participants (297 out of 924, 32%) were not physically active at moderate to vigorous intensity for at least 30 minutes per day, and 435 out of 924 (47%) had insufficient fruit and vegetable intake. The randomization was not completely successful in creating comparable groups. There was a difference for fruit intake at baseline, with more participants in the intervention meeting the guideline (intervention: 57%, reference: 50%).

### Initial Participation

Of the 183 non-participants responding to the questionnaire for non-participants, most gave ‘I am healthy’ (41%) as their reason for not participating in the program; followed by ‘other reasons’ (34%), of which most were practical reasons such as a lack of time, forgotten to subscribe, or unaware of the existence of the program; 13% of the non-participants would like to keep private life and work separated; and 19% of the non-participants preferred to arrange health promotion activities themselves. Most participants (86%) mentioned ‘curious about my health’ as their most important reason to participate.

Employees aged 30 years and older were more likely to participate in the program (ORs between 1.57 and 2.25). Workers with a low intention to increase their PA level were less likely to participate (OR=0.60, 95%CI 0.43-0.85).

**Table 1:** Differences between participants (n=924) and a sample of non-participants (n=183) in a workplace health promotion program.

Characteristics		Participants n (%)	Non-participants n (%)	Initial participation univariate analyses OR (95%CI)
**Demographics**				
	Male gender	450 (49)	62 (34)	1.17 (0.81-1.70)
	Age <30 years	128 (14)	43 (24)^c^	1.00
	Age 30-39 years	253 (27)	46 (25)	1.57 (0.96-2.57)
	Age 40-49 years	277 (30)	39 (22)	2.25^a^ (1.36-3.72)
	Age ≥50 years	266 (29)	53 (29)	1.61 (0.99-2.62)
	Education high	414 (45)	102 (56)	1.00
	Education intermediate	306 (33)	49 (27)	1.15 (0.78-1.70)
	Education low	204 (22)	32 (18)	1.05 (0.66-1.68)
	Non-Dutch ethnicity	151 (16)	29 (16)	1.04 (0.66-1.63)
	Unmarried / not cohabiting	222 (24)	41 (22)	1.20 (0.81-1.77)
**Lifestyle factors**				
	<30min/day PA	297 (32)	58 (32)	1.01 (0.71-1.44)
	<3x20min/day vigorous PA	652 (71)	128 (70)	1.15 (0.80-1.65)
	<400g/day fruit and vegetables	435 (47)	n/a^d^	n/a
	Current smoker	165 (18)	28 (15)	1.08 (0.69-1.70)
	Excessive alcohol consumption	24 (3)	5 (3)	0.98 (0.36-2.67)
	BMI^b^ < 25 kg/m^2^	460 (57)	119 (65)	1.00
	25 kg/m^2^ ≤ BMI^b^ < 30	277 (34)	58 (32)	1.11 (0.69-1.44)
	BMI≥30 kg/m^2^	70 (9)	6 (3)	2.24 (0.93-5.42)
**Social cognitive factors for physical activity**				
	Poor attitude	464 (50)	87 (47)	1.24 (0.89-1.72)
	Low support from family	772 (84)	166 (90)	0.63 (0.37-1.08)
	Low self-efficacy	214 (23)	33 (18)	1.48 (0.97-2.25)
	Low intention	478 (52)	121 (66)	0.60^a^ (0.43-0.85)
**Social cognitive factors for fruit and vegetable intake**				
	Poor attitude	280 (30)	n/a	n/a
	Low support family	784 (85)	n/a	n/a
	Low self-efficacy	167 (18)	n/a	n/a
	Low intention	180 (20)	n/a	n/a
**Health Indicators**				
	Poor/moderate general health	58 (6)	15 (8)	0.83 (0.45-1.52)
	Elevated blood pressure^b^	258 (32)	n/a	n/a
	Poor predicted Vo_2_max^b^	267 (36)	n/a	n/a
	Elevated total cholesterol^b^	338(42)	n/a	n/a
**Work-related factors**				
	Physical job demands	148 (16)	42 (24)	0.84 (0.56-1.56)
**Health Check participation**		811 (88)	n/a	n/a
**Intervention group**		456 (49)	n/a	n/a

^a^ *P*<0.05, analyses adjusted for company

^b^BMI, blood pressure, maximum oxygen uptake, and cholesterol level are only available for the participants in the physical health check (n=807)

^c^ n=2 missing for age of non-participants.

^d^n/a: not available

### Sustained Participation: Questionnaire and Health Check

After 1 year, 666 out of 924 participants (72%) filled out the questionnaire, and 558 out of 924 (60%) filled out the 2-year follow-up questionnaire to obtain feedback on their lifestyle. In the intervention group, 68% filled out the first follow-up questionnaire and 58% the second follow-up questionnaire. In the reference group this was respectively 76% and 63%.

As shown in Table 2, older employees were more likely to sustain their participation at follow-up, while employees with a non-Dutch ethnicity were less likely to sustain their participation. In the univariate analysis married/cohabiting participants were more likely to keep participating, but after adjustment for age, lifestyle and health indicators the association diminished and did not remain statistically significant (OR_1yr_= 1.34, 95%CI 0.87-2.07; OR_2yr_=0.94, 95%CI 0.63-1.40).

Smokers and participants with a poor predicted maximum oxygen uptake were less likely to sustain their participation. Insufficient fruit and vegetable intake was also associated with reduced participation in the follow-up measurements, but this association did not remain statistically significant after adjustment for the predicted maximum oxygen uptake (OR_1yr_=0.87, 95%CI 0.60-1.26, OR_2yr_=0.87, 95%CI 0.63-1.20). Participants in the intervention condition were less likely to participate again after one year compared with the reference condition. This relation was not apparent in the analysis for participation at 2-year follow-up.

Participants with a low intention to change their PA level (OR_1yr_=1.69, 95%CI 1.26-2.27; OR_2yr_ 1.40, 95%CI 1.07-1.83) or fruit and vegetable intake (OR_1yr_=2.06, 95%CI 1.45-2.92; OR_2yr_ =1.93, 95%CI 1.38-2.70) were more likely to participate at 1- and 2-yr follow-up. Workers with low social support (PA: OR=0.44, 95%CI 0.30-0.63, FV: OR=0.46, 95%CI 0.31-0.68) and low self-efficacy (PA: OR=0.16, 95%CI 0.11-0.23, FV: OR=0.38, 95%CI 0.22-0.66) were less likely to have the intention to change their behavior (not in table).

Most employees participating in the 2^nd^ follow-up questionnaire also participated in the 2^nd^ physical health check (65%), and, except insufficient fruit and vegetable intake, similar determinants were found as for sustained questionnaire participation. Older participants (OR_40-49yr_=1.99, 95%CI 1.19-3.33; OR_50+yr_=1.74, 95%CI 1.01-2.9) and those with a Dutch ethnicity (OR­_­_=1.59, 95%CI 1.02-2.44) were statistically significantly more likely to participate in the physical health check. Employees with a low intention to change their behavior were more likely to participate in the follow-up health check (PA: OR=1.28, 95%CI 0.97-1.70, FV: OR=1.47, 95%CI 1.01-2.15) (not in table).

**Table 2:** Determinants of sustained participation after 1 and 2 years in a workplace health promotion program (n=924).

Characteristics		1 year follow-up participation		2 year follow-up participation	
		univariate	multivariate	univariate	multivariate
		OR (95%CI)	OR (95%CI)	OR (95%CI)	OR (95%CI)
**Demographics**					
	Male gender	1.42^a^ (1.03-1.97)	1.04 (0.70-1.55)	1.41^a^ (1.05-1.90)	1.15 (0.81-1.64)
	Age <30 years	1.00	1.00	1.00	1.00
	Age 30-39 years	1.51 (0.97-2.36)	1.47 (0.70-1.55)	1.33 (0.86-2.05)	1.39 (0.83-2.32)
	Age 40-49 years	2.52^a^ (1.59-4.00)	2.36^a^ (1.35-4.12)	2.29^a^ (1.48-3.54)	2.08^a^ (1.25-3.47)
	Age ≥50 years	3.01^a^ (1.86-4.86)	2.38^a^ (1.30-4.33)	2.89^a^ (1.84-4.55)	2.47^a^ (1.43-4.26)
	Education high	1.00	1.00	1.00	1.00
	Education intermediate	1.14 (0.80-1.62)		0.98 (0.71-1.34)	
	Education low	0.77 (0.52-1.14)		0.72 (0.50-1.03)	
	Non-Dutch ethnicity	0.62^a^ (0.42-0.89)	0.77 (0.48-1.22)	0.47^a^ (0.33-0.67)	0.51^a^ (0.34-0.78)
	Unmarried / not cohabiting	0.61^a^ (0.44-0.85)		0.73^a^ (0.53-0.99)	
**Lifestyle factors**					
	<30min/day PA	0.75 (0.56-1.02)		0.83 (0.63-1.11)	
	<3x 20min/day vigorous PA	1.03 (0.74-1.42)		1.14 (0.85-1.53)	
	<400gr/day fruit & vegetables	0.65^a^ (0.49-0.88)		0.73^a^ (0.56-0.96)	
	Current smoker	0.53^a^ (0.37-0.75)	0.52^a^ (0.33-0.82)	0.51^a^ (0.36-0.72)	0.54^a^ (0.35-0.82)
	Excessive alcohol intake	1.29 (0.50-3.33)		0.93 (0.41-2.13)	
	BMI < 25 kg/m^2^	1.00	1.00	1.00	1.00
	25 kg/m^2^ ≤ BMI < 30	1.17 (0.81-1.68)		0.98 (0.71-1.35)	
	BMI ≥ 30 kg/m^2^	1.29 (0.69-2.42)		0.90 (0.53-1.53)	
**Health indicators^a^**					
	Elevated blood pressure	1.22 (0.85-1.76)		0.95 (0.69-1.31)	
	Poor predicted Vo_2_max	0.54^a^ (0.38-0.76)	0.56^a^ (0.39-0.81)	0.66^a^ (0.48-0.91)	0.76 (0.55-1.06)
	Elevated total cholesterol	1.07 (0.77-1.49)		1.35^a^ (1.00-1.82)	
	Decreased general health	0.71 (0.40-1.25)		0.49^a^ (0.28-0.84)	
**Work-related factors**					
	Physical job demands	0.71 (0.48-1.05)		0.75 (0.52-1.09)	
**Intervention group**		0.63^a^ (0.47-0.85)	0.56^a^ (0.39-0.81)	0.85 (0.65-1.12)	0.92 (0.67-1.28)

* *P*<0.05, analyses adjusted for company

^a^ BMI, blood pressure, maximum oxygen uptake, and cholesterol level are only available for the participants in the physical health check (n=807)

### Sustained Participation: Website Use

Six percent of the participants did not visit the website throughout the study period, 18% visited the website once, 13% twice, and 64% three times or more. The range of website visits during the whole 2-year study period was 0–46, with a median of 3 visits (interquartile range: 2-6 visits). Participants visited the website more frequently in the first months after the questionnaires (Table 3). In the period 4-12 months after baseline, participants in the intervention condition, who received monthly email messages during this period, were more likely to visit the website (OR=5.88, 95%CI 3.75-9.20, adjusted for company). 

As shown in Table 4, smokers were less likely to visit the website than non-smokers. Participants with a low intention to increase their fruit and vegetable intake (1-3 months: 1.73, 95%CI 1.31-2.27; 4-12 months: OR=1.47, 95%CI 0.92-2.36) were more likely to visit the website (not in table).

**Table 3:** Website visits and use of different website functionalities throughout the study in the reference (R) and intervention (I) program.

Month	Study population		Website visits		Viewed advice physical activity		Viewed advice fruit and vegetables		Self- monitor	fat FFQ	Asked question
	R	I	R	I	R	I	R	I	I only	I only	I only
	n	n	%	%	%	%	%	%	%	%	%
1-3^b^	386	412	74	71	30	27	30	25	7	n/a	0
4-12	468	456	6	27^a^	2	3	2	3	6	15	3
13-15^c^	385	344	76^a^	66	23	18	26^a^	17	1	n/a	1
16-24^c^	385	344	2	3	1	0	1	0	0	n/a	0
25-28^d^	294	264	89	85	18^a^	11	19^a^	10	2	n/a	1

^a^ Chi-square, *P*<0.05

^b^ for one company (executive branch of government) no information was available for website use in the first three months of the study.

^c^ participants not responding to the first and second follow-up questionnaire were considered as drop-outs and not included in the analysis for 13-24 months.

^d^ participants not responding to the second follow-up questionnaire were considered as drop-outs and not included in the analysis for 25-28 months.

**Table 4:** Determinants of website use of a workplace health promotion program.

Characteristics		Website use month 1-3 after invitations to fill out questionnaire (n=630)^c^		Website use month 4-12 after invitations to fill out questionnaire (n=729)	
		univariate	multivariate	univariate	multivariate
		OR (95%CI)	OR (95%CI)	OR (95%CI)	OR (95%CI)
**Demographics**					
	Male gender	0.88 (0.71-1.11)	0.87 (0.69-1.09)	1.09 (0.78-1.52)	
	Age <30 years	1.00	1.00	1.00	1.00
	Age 30-39 years	1.05 (0.74-1.51)	1.06 (0.74-1.52)	0.90 (0.50-1.61)	0.85 (0.47-2.20)
	Age 40-49 years	1.31 (0.92-1.88)	1.32 (0.92-1.89)	1.15 (0.66-2.00)	1.12 (0.64-1.98)
	Age ≥50 years	1.14 (0.80-1.63)	1.18 (0.82-1.70)	1.25 (0.72-2.18)	1.24 (0.70-1.52)
	Education high	1.00		1.00	
	Education intermediate	0.86 (0.67-1.11)		0.96 (0.66-1.40)	
	Education low	0.73^a^ (0.54-0.97)		0.83 (0.53-1.30)	
	Non-Dutch ethnicity	0.96 (0.70-1.31)		0.82 (0.51-1.33)	
	Unmarried / not cohabiting	1.06 (0.81-1.38)		0.86 (0.58-1.29)	
**Lifestyle factors**					
	<30min/day PA	1.03 (0.81-1.31)		1.04 (0.73-1.48)	
	<3x 20min/day vigorous PA	1.11 (0.87-1.41)		0.83 (0.59-1.18)	
	<400gr/day fruit & vegetables	0.86 (0.69-1.07)		0.83 (0.59-1.16)	
	Current smoker	0.71^a^ (0.53-0.95)	0.72^a^ (0.54-0.96)	0.64 (0.39-1.04)	0.66 (0.40-1.08)
	Excessive alcohol intake	1.01 (0.50-2.05)		0.87 (0.29-2.59)	
	BMI < 25 kg/m^2^	1.00		1.00	
	25 kg/m^2^ ≤ BMI < 30	1.08 (0.84-1.40)		1.03 (0.56-1.91)	
	BMI ≥ 30 kg/m^2^	1.19 (0.76-1.86)		0.96 (0.52-1.77)	
**Health indicators**					
	Elevated blood pressure	1.13 (0.87-1.46)		1.13 (0.79-1.61)	
	Poor predicted Vo_2_max	0.75 (0.53-1.08)		0.99 (0.59-1.65)	
	Elevated total cholesterol	1.04 (0.81-1.32)		0.84 (0.59-1.18)	
	Decreased general health	0.83 (0.51-1.34)		0.97 (0.48-1.96)	
**Work-related factors**					
	Physical job demands	1.00 (0.73-1.36)	0.99 (0.71-1.36)	0.57^a^ (0.33-0.97)	0.59 (0.34-1.02)

^a^ *P*<0.05, analyses adjusted for company

^b^ BMI, blood pressure, maximum oxygen uptake, and cholesterol level are only available for the participants in the physical health check (n=807)

^c^ for one company (executive branch of government) no information was available for website use in the first three months of the study.

## Discussion

Modest initial participation and a high attrition in a health promotion program were found in the current study. Employees aged 30 years and older were more likely to start in the program as well as to sustain their participation. Lifestyle and health indicators were not related to initial participation, but did play a role in sustained participation as well as in visiting the website throughout the study period. Workers with a low intention to change their PA level were less likely to start, but once enrolled they were more likely to sustain participation and use the website.

### Participation

Previous studies reported low participation and high levels of attrition in Internet-delivered health promotion programs [4,8-10]. In a systematic review, a median reach of 33% (95% CI 25–42%) was found in workplace health promotion programs [1]. In our study, three companies restricted the maximum number of employees allowed to enroll, leading to an artificially lower participation level. Without these companies, a mean initial participation level of 43% was established. Once enrolled, 72% also participated in the 1^st^ follow-up, and 60% in the 2^nd^ follow-up measurement. This is in agreement with a systematic review reporting that the majority of Internet-delivered weight loss programs had less than 80% retention [5].

### Determinants of Participation

In a systematic review studying initial participation in workplace health promotion, no major differences in lifestyle and health indicators were identified between those who participated and those that did not [1]. With our focus on workers, a relatively healthy group was reached, since workers are in better health than unemployed individuals [33]. In general, the employees who participated in our study had a quite similar lifestyle and health as non-participants. Although non-significant, obese workers were more likely to enroll in the study. This is in accordance with other recent studies [10,16] which have argued that this might be due to the non-stigmatizing way of addressing body weight through the Internet.

In line with other studies [10,13,14,16,17,19], we found that older employees started more often and sustained their participation during the follow-up measurements. A recent study on a workplace health promotion program also reported increased participation among older workers [34], which is a promising finding regarding the higher risk of cardiovascular diseases at older age, and employers’ focus on keeping the ageing workforce healthy. However, although we found that older employees visited the website more often, they did not use the website components more often compared with younger workers.

It is remarkable that employees with a low intention to change their behavior were less likely to enroll in the study, but once participating, those with a low intention were more likely to sustain at follow-up, and to visit the website throughout the study period. This might indicate that the program is appealing for employees with a low intention to change their behavior. Alternatively, it might also indicate that those participants who intend to change their behavior do not need the program to get into action. To our knowledge, only one previous study provided information on the role of intention to change in participation [13]. They did report that workers with a positive health motivation were more likely to continue website use, but did not find an influence of intention to increase PA and participation. Measuring intention to change in other studies could provide more insight into the role of intention in participation, and might help to find out what program content facilitates reach and sustained participation for those with a low intention to change.

Previous studies have presented contradictory results concerning the relationship between lifestyle and health indicators and sustained participation, with some studies describing elevated participation among those who need it most [10,14] and other studies among those who are already healthy or engage in a healthy lifestyle [10,19,21]. We found a consistently lower sustained participation among smokers and employees with a low cardiorespiratory fitness. These associations between unhealthy lifestyle behaviors and decreased participation might reduce the effectiveness of primary preventive interventions.

### Website Functionalities

Previous studies reported high attrition in Internet-delivered health promotion programs [8,10]. We also found a reduction in website visitors throughout the study period, with peaks in the months after the invitations to fill out the questionnaires. Between the questionnaire invitations, the companies did too little to communicate about the program to their personnel. Therefore, the embedding of the program in the organizations was limited.

Participants in the intervention group received monthly email messages during the first study year, and 27% visited the website compared with 6% in the reference group during this period. These monthly email messages seem to work as a prompt for website visits, but might have had a negative influence on sustained participation, since less participants in the intervention group filled out the first follow-up questionnaire. In the second year after baseline, no monthly email messages were sent, and at the end of the second year there was no statistically significant difference between the reference and intervention condition in both follow-up participation and website visit. A possible explanation for the lower response among intervention participants at the first follow-up could be that they felt less need to fill out the questionnaire to obtain feedback, because they already received information in the monthly messages and self-monitors throughout the year. In a systematic review it was concluded that the use of periodic prompts can be effective in behavior change interventions [35]. However, the optimal frequency and structure of such prompts is still unknown.

In accordance with previous studies [36], users appear not to be optimally utilizing key aspects of the intervention. A qualitative systematic review suggested that only peer support, counselor support, email/phone contact with visitors, and updates of the intervention website were related to better exposure to health promotion through the Internet [37]. In the current study, only a minority of the employees who filled out the questionnaires also read the subsequent advice on PA and fruit and vegetable intake, and the number of participants using self-monitors on a regular basis and asking questions to professionals was limited. This is in contrast with the wishes of potential users as identified in focus group interviews [38]. It could be questioned whether self-monitoring and the possibility of asking questions fit with the wishes of our target group. In addition, there are numerous websites freely available with self-monitor functions and possibilities to obtain personal feedback on lifestyle and health, which might reduce employees’ need of another website. Participants could use the website at their own discretion, which might have led to lower use, making this minimal effort intervention not enough to elicit program compliance.

### Limitations

This study has some limitations. First, the 25% response to the questionnaire for non-participants was low, and might hamper comparison between responders and non-responders. Second, the measures for website use did not provide any information as to what extent the participants actually read the available information or how much time they spent on the website. In addition, reasons for drop-out are unknown. Therefore, we do not know whether individuals stop participating because they do not need the program anymore, because they are dissatisfied with the program, or because of another reason. Furthermore, because of technical problems no data on website use were collected for 99 participants during the first three study months. Third, there was no information available on the use of other lifestyle-related websites simultaneous with our website. However, we do not expect a difference between the intervention and reference condition concerning the use of other websites. Last, walking was not included in moderate to vigorous PA. Because of the many hours individuals indicated they spent walking, we hypothesize that work-related walking played a major role in over-reporting physical activity. Since work-related walking is, in most cases, not brisk walking, we decided to exclude the question.

### Conclusion

Modest initial participation and high attrition in program use were found in the current study. Workers with a low intention to increase their PA level were less likely to enroll in the intervention, but once participating they were more likely to sustain their participation. Lifestyle and health indicators were not related to initial participation, but those with an unhealthier lifestyle were less likely to sustain their participation. This might influence program effectiveness as those who can benefit most are limitedly utilizing health promotion programs. Regular email messages prompted website use, but the use of important Web-based tools was limited. There is a need for more appealing techniques to enhance retention and to keep those individuals who need it most attracted to the program.

## Abbreviations

BMI: body mass index
CI: confidence interval
GEE: general estimating equations
n/a: not available
OR: odds ratio
PA: Physical activity
